# The Influence of Micropatterned Surfaces on Platelet Adhesion to Reduce the Risk of Thrombus Formation in Left Ventricular Assist Devices

**DOI:** 10.1007/s10439-026-04108-7

**Published:** 2026-03-27

**Authors:** Marta Bonora, Stjepan Perak, Sonja Kopp, Josu Garcia Morales, Richard Benauer, Markus Lunzer, Michael Mühlberger, Teresa Ruthmeier, Barbara Messner, Michael Bernhard Fischer, Francesco Moscato

**Affiliations:** 1 Center for Medical Physics and Biomedical Engineering, Medical University of Vienna, Vienna, Austria; 2 Ludwig Boltzmann Institute for Cardiovascular Research, Vienna, Austria; 3 UpNano GmbH, Vienna, Austria; 4 PROFACTOR GmbH, Steyr, Austria; 5 Bionic surface technologies GmbH, Graz, Austria; 6 Department of Cardiac and Thoracic Aortic Surgery, Medical University of Vienna, Vienna, Austria; 7 Department of Transfusion Medicine and Cell Therapy, Medical University of Vienna, Vienna, Austria; 8 Department for Biomedical Research, University for Continuing Education Krems, Krems, Austria; 9 Austrian Cluster for Tissue Regeneration, Vienna, Austria

**Keywords:** Left ventricular assist devices (LVAD), Platelet adhesion, Micron/nano 3D printing, Micropatterning, Thrombosis

## Abstract

**Purpose:**

Surface micropatterning is being explored as a strategy to mitigate thrombus formation and reduce long-term anticoagulation requirements in left ventricular assist devices (LVADs). This study investigated whether specific micro-topographies can modulate platelet deposition under LVAD supraphysiological wall shear stress (WSS) conditions.

**Materials and Methods:**

A custom microfluidic platform was developed to generate a linear WSS gradient from 16 to 130 Pa. Microchannels were patterned with reverse cones and hemispheres in small (1–3 *µ*m) and large (3–9 *µ*m) sizes using two-photon polymerization and nanoimprinting. Human blood was perfused through the microchannels, and platelet deposition was quantified over time as the area coverage ratio (*A*ₚₗₜ/*A*ₜₒₜ) and area under the curve (AUC). Platelet detachment events were counted as an indicator of thrombus stability, and computational simulations supported the interpretation of local shear conditions.

**Results:**

Consistent trends emerged, although no statistical differences were observed relative to flat controls. *A*ₚₗₜ/*A*ₜₒₜ increased with WSS for all surfaces. At 16 Pa, small and large cones reduced platelet adhesion by approximately 84 and 98%, respectively, compared to flat controls. At 49 Pa, the reduction was about 95% for small cones and 80% for large cones. Conical geometries also promoted platelet washout at higher WSS. Small hemispherical features showed more than 50% lower platelet adhesion than flat surfaces for WSS > 16 Pa, with limited thrombus growth.

**Conclusion:**

Conical micropatterns may be most effective at limiting platelet adhesion at lower WSS, while small hemispheres may perform better at higher WSS. These trends suggest a surface–shear interaction that warrants further investigation for LVAD surface optimization.

**Supplementary Information:**

The online version contains supplementary material available at https://doi.org/10.1007/s10439-026-04108-7.

## Introduction

Supraphysiological wall shear stress (WSS) (>10 Pa) in centrifugal left ventricular assist devices (LVADs) [1] leads to platelet activation followed by adhesion and aggregation on artificial surfaces, ultimately resulting in thrombus formation, which may obstruct blood flow in situ or cause thromboembolic events [2, 3]. Mechanical stimuli can directly activate platelets which can lead to aggregation without the need for chemical agonists [4]. For extreme high WSS conditions, the aggregation of platelets can occur even in the absence of prior activation [5]. Not only the magnitude but also the gradient of WSS experienced by blood cells plays a key role in modulating platelet adhesion acting as a powerful agonist of the activation [6–8]. Formation of thrombi during mechanical left ventricular support leads to serious consequences for patients [2]. Third-generation LVADs, such as the HeartMate 3 and HeartWare VAD, have achieved 2-year survival rates above 80%, comparable to heart transplantation. However, device-associated thrombosis and thromboembolic complications still occur [2, 9, 10]. To prevent uncontrolled coagulation, lifelong anticoagulation therapy, typically Warfarin, is required. This treatment demands regular monitoring, as incorrect dosing increases the risk of thromboembolic events and, more frequently, severe bleeding [11]. Despite strict therapy, thrombosis is not completely prevented [12], and the need for continuous anticoagulation significantly reduces patient quality of life [13]. Emerging therapies show promising early results, but robust clinical evidence is still lacking [10]. Therefore, there is a strong need for alternative or complementary strategies that passively reduce thrombus formation. One promising approach is the engineering of surface micro- and nano-topographies designed to limit blood cell adhesion and activation at the material interface. Surface micropatterning increases the hydrophobicity [14, 15] and reduces blood–surface interactions and consequently the platelet adhesion [16, 17]. Under dynamic conditions, in physiological regime, micropatterned surfaces were shown to decrease platelet adhesion compared to an unstructured surface since platelets come only in contact with the tips of the microstructures decreasing the overall contact with the surface [18] and the adhesion is hindered by the heterogeneous WSS distribution [19]. In contrast, the platelet adhesion on a surface with a random roughness increases compared to an unstructured surface [20, 21], and microstructures exceeding in height can disturb blood flow creating vortices and platelet accumulation [3], suggesting that geometry, arrangement, and dimensions of microstructures are central in modulating platelet interactions. Previous findings present three limitations: (1) all of the studies were conducted under static or physiological WSS conditions, underestimating the supraphysiological shear effects in presence of LVADs; (2) in real conditions, platelets are exposed to a wide range of non-uniform WSS; and (3) rationales behind the design of the micropatterning are often nonspecific for this application. The main aim of this study is to investigate, for the first time, the effect of micropatterned surfaces under supraphysiological WSS conditions relevant to centrifugal blood pumps. Furthermore, a novel micropatterning manufacturing process with newly designed geometries was employed. Hemispheres and reverse cones were selected as geometries for micropatterning of a surface, which was integrated into a microfluidic device. The variable height of the microchannel generates a WSS gradient along its length, reproducing a range of WSS values comparable to those found in LVADs. This design specifically addresses the large fraction of blood volume within a newly designed pediatric pump that is exposed to WSS within the selected range [22]. Along the microchannel, four distinct WSS regions were defined from the inlet to the outlet: 130, 114, 49, and 16 Pa. Mepacrine-labeled platelets in whole blood diluted 1:1 (v/v) with phosphate-buffered saline (PBS) were perfused through the microchannel, and surface adhesion was captured in frames over time. The microchannel masters were fabricated by two-photon polymerization (2PP) 3D printing [23, 24] and replicated using nanoimprinting. This strategy was chosen to reduce platelet adhesion, while also promoting more frequent detachment of small aggregates compared to flat controls.

## Materials and Methods

### Design of the Microchannel

**Fig. 1:**
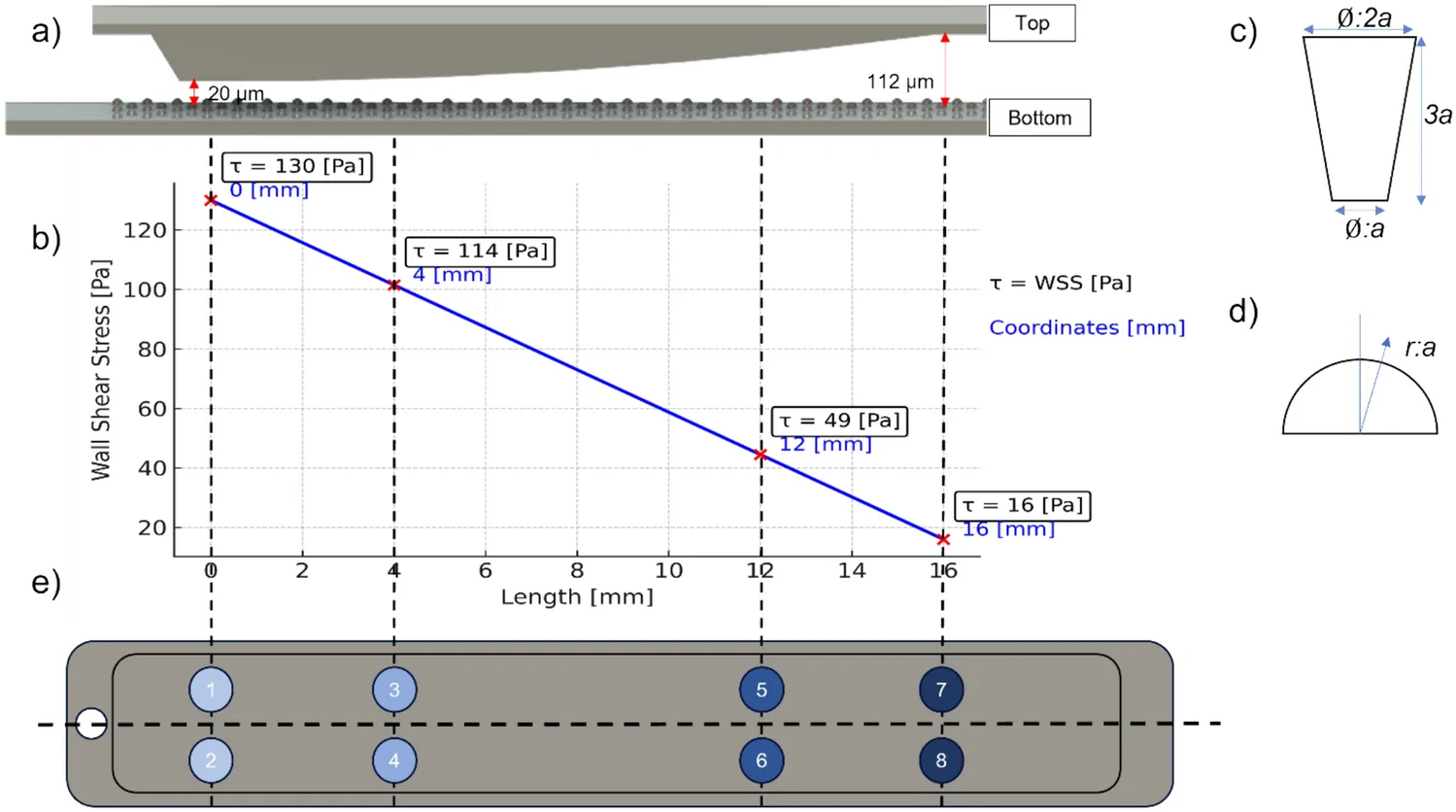
**a** Sketch of a microchannel in side view (not to scale); **b** WSS linear profile plotted along the channel length, induced by the ramp geometry; **c** cross-section of the reverse cone microstructure; **d** cross-section of the hemisphere microstructure; **e** microchannel top view indicating the four fixed positions along the channel length for image acquisition. Fusion 360 (Autodesk) and Microsoft PowerPoint were used to create the artwork

The microchannel is composed of a top and a bottom layer, Fig. 1a. The top layer is designed with a variable height (gap from 20 to 112 *µ*m) aimed at creating a linear decreasing WSS within the channel during the blood perfusion, Fig. 1b. The bottom layer is patterned with microstructures along the entire length of the microchannel (CAD design and details of the channel in Online Resource 1, Fig. S1). To obtain the ramp profile and the linear decreasing WSS, the channel height has been calculated using Eq. 1:

1 $$H\left( x \right) = \sqrt {\frac{1}{{\frac{1}{{Hl^{2} }} + \left( {\frac{1}{{H0^{2} }} - \frac{1}{{Hl^{2} }}} \right)* \frac{X}{L}}}}$$

where $H\left(x\right)$ is the height of the channel with respect to the length of the channel x, $Hl=H\left(x=L\right), {H}_{0}=H\left(x=0\right),$ and L is the total length of the channel. The equation is obtained considering Eqs. 2 and 3:

2 $$\tau = \tau_{\max } + \left( {\tau_{\min } - \tau_{\max } } \right)* \frac{x}{L}$$

where $$\tau_{\max } = \tau \left( {x = 0} \right) and \tau_{\min } = \tau \left( {x = L} \right)$$

3 $$\tau = \frac{6*Q*\mu }{{W*H\left( X \right)^{2} }}$$

where τ is the WSS for a channel with a rectangular cross-section area, Q is the flow rate, μ is the viscosity, W is the width of the channel (2 mm), and $H\left(x\right)$ is the height.

The microstructure geometries are reverse cones (*C*) and hemispheres (*H*) in two sizes, small (*S*) and large (*L*). Dimensions of *C* and *H* are shown in Fig. 1c and d, where *S* and *L* structures are obtained, respectively, for *a* = 1 and *a* = 3 *µ*m. Microstructures are arranged in a staggered configuration and spaced 3 a apart, center to center. Reverse cone microstructures were designed to promote the maintenance of hydrophobicity through re-entrant geometries, in accordance with the Cassie–Baxter wetting regime. Hemispherical microstructures were selected based on bioinspired microstructures mimicking endothelial cell morphology [19]. A staggered arrangement was adopted to minimize the formation of protected niches that could favor platelet accumulation. Feature dimensions close to the characteristic platelet size were selected to hinder platelet infiltration between adjacent microstructures and to prevent complete platelet spreading over individual features. More details are summarized in our previous publication [25].

### Computational Simulation of the Microchannel with the Ramp

The microchannel ramp design was validated by CFD (ANSYS Fluent). A 2 mm inlet was added to stabilize the velocity profile (Online Resource 1, Fig. S2a), and boundary conditions were set: velocity inlet, pressure outlet, and no-slip walls. A structured mesh of approximately 1.25 million hexahedral elements was generated in ICEM CFD (Online Resource 1, Fig. S2b). The Reynolds number confirmed laminar flow, and blood was modeled as a Newtonian fluid. WSS was estimated using a constant viscosity of 3.5 cP; this approximation is based on hematocrit estimation [26] and accounts for the dilution applied and the temperature at which the experiment is performed. WSS served as the validation parameter, and the four operating points were selected and adjusted based on the simulation (Fig. 1b and e).

### Computational Simulation of the Microstructures

To simulate the effect of the microstructures on flow, a periodic domain was constructed to limit computational cost, and simulations were performed with ANSYS Fluent at three operating points at the entrance, halfway, and endpoint of the microchannel, corresponding to channel heights of 20, 30, and 112 *µ*m. The periodic domain was implemented with periodic boundary conditions, whereby inlet, outlet, and lateral faces were coupled to enforce identical flow fields (Online Resource 1, Fig. S3). A polyhedral mesh was selected for each one of the structures at every operating point (Online Resource 1, Fig. S4). The size of the meshes was in the range of 3 to 10 million cells, with the biggest meshes corresponding to the smallest structures. The simulation setup followed the same assumptions used for the microchannel.

### Fabrication of the Microchannel

The microchannels were manufactured from 2PP printed master structures, which were replicated by nanoimprinting (microstructured bottom plate) and polydimethylsiloxane (PDMS) replication (top ramp). As the bottom plate was replicated by nanoimprinting, the master was a positive of the final replicate, whereas the master for the top ramp was produced as a negative for direct PDMS casting.

### 2-Photon Polymerization (2PP) of Microchannel Masters

**Fig. 2:**
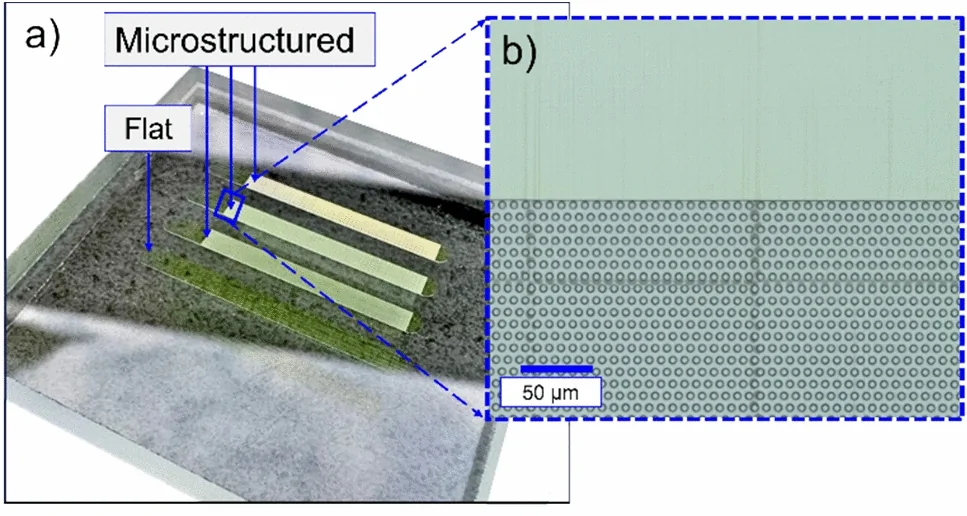
**a** Photograph of an array of four 2PP 3D printed bottom layer masters, each being 23 mm long and 2 mm wide, and micropatterned with hemispheres; **b** detailed light microscopy image (VHX-7000, Keyence, Japan) of the large hemispheres

The ramped top and the micropatterned bottom masters for the microchannel were micromanufactured using the 2PP 3D printing system NanoOne 1000 (UpNano GmbH, Austria) in vat printing mode [27]. The resin vat, equipped with a high-precision glass window, is positioned above the objective, preventing contact with the resin and preserving the focusing power of the objective. The part is printed layer by layer and drawn out of the vat while maintaining a constant distance between the current print layer and the objective. This approach ensures consistently high precision throughout the printing process. The nonlinear nature of the excitation allows a continuous liquid film to persist between the part and the glass window, enabling a fast-printing process. Submicron precision was achieved using UpNano’s patented *Adaptive Resolution* 2PP technology, which allows dynamic switching of voxel size between high-resolution and high-throughput printing regions to reduce print time without compromising localized detail. The bottom masters patterned with *C* and *H* in size *S* and *L* and the flat reference channel (Fig. 2a and b) were printed with UpBrix2 resin (UpNano GmbH, Austria) using a high-resolution 40 × oil-immersion objective (NA 1.4, Olympus, Japan). Top-layer masters were printed with UpPhoto resin (UpNano GmbH, Austria, Online Resource 1, Fig. S11) using a 10 × air objective (NA 0.4, Olympus, Japan). In both cases, methacrylated borosilicate glass (40 × 40 × 5.5 mm^3^) was used as the substrate [28].

### Nanoimprinting

Nanoimprinting with OrmoComp (micro resist technology GmbH, Germany) was employed to replicate the microstructured master onto microscope glass slides (SuperFrost Plus, Epredia, NH USA), while the top of the microfluidic chips was fabricated in PDMS Sylgard 184. Prior to imprinting, the 2PP masters were baked at 40 °C for 48 h and subsequently coated with an anti-adhesive layer (BGL-GZ-96, ProFactor GmbH). UV curing was performed at 365 nm with an intensity of 100 mW/cm^2^ and a dose of 30 J/cm^2^. To obtain residual-layer-free imprints, a process based on micro-molding in capillaries (MIMIC) [29] was applied, which has been demonstrated to suit for this purpose in previous yet unpublished works. The top layer was produced by casting PDMS, followed by curing at 40 °C overnight on a hotplate to minimize shrinkage [30]. For final device assembly, both the residual-layer-free imprinted glass slide and the PDMS top ramp were plasma activated (Diener Nano, Diener Electronics GmbH, Germany), then aligned manually into contact and left to rest for 15 h before use. More info on the nanoimprinting is in the supplementary Information in the dedicated section (Online Resource 1, “Nanoimprinting”).

### Experimental Section

#### Blood Collection

Whole blood was collected in NH sodium heparin ~ 16 IU/mL of blood (Vacuette tubes, 9 mL, green cap; ref. 455051, Greiner Bio-One, Austria) from patients with hemochromatosis at the Department of Transfusion Medicine and Cell Therapy, General Hospital of Vienna, and from healthy volunteers at the Center for Physiology and Pharmacology, Medical University of Vienna. The study was approved by the Ethics Committee of the Medical University of Vienna (EK Nr. 1548/2020, EK Nr. 1189/2024) after written informed consent was obtained.

#### Blood Preparation

Collected blood was diluted 1:1 (v/v) with PBS (pH 7.4; Gibco, Thermo Fisher Scientific) and maintained at 37 °C until perfusion in the microchannels [31], while experiments were performed at room temperature. Mepacrine fluorescent dye (Mepacrine or Quinacrine Dihydrochloride, Sigma-Aldrich, Merck KGaA, Germany) was added to the blood–PBS solution 20 min before perfusion, yielding a final concentration of 4 *µ*m. All blood samples were used within 4 h of collection.

#### Experimental Setup

In each experiment, five microchannels, small cones (SC), large cones (LC), small hemispheres (SH), large hemispheres (LH), and flat control without micropatterning were perfused with the same blood, ensuring identical biological conditions across all tested geometries. Channel inlets were equipped with in-house-built connectors and connected via silicone tubing (Ø 0.8 mm; Ibidi GmbH, Germany) to a syringe pump (11 Pico Plus Elite, Harvard Apparatus, USA), which perfused each channel with 6 mL of blood at a flow rate of 0.6 mL/min for 10 min. This required a total volume of 30 mL per experiment. Accordingly, three blood collection tubes were sufficient to complete each experiment, while a fourth tube was collected as backup. Microfluidic chips containing microchannels were mounted on an inverted fluorescence microscope (NA 0.7, Olympus IX83, Japan) equipped with a camera (ORCA-Flash4.0, Hamamatsu Photonics, Japan). Along the x-axis of the microchannel, four positions corresponding to distinct WSS values were identified. At each WSS position, two equidistant points were selected, resulting in eight fixed acquisition points per channel (Fig. 1e) with a WSS deviation of ± 4.8 Pa within the evaluation area (590 × 590 *µ*m^2^). The syringe pump was started to initiate blood flow, and image acquisition began immediately once blood reached the microchannel inlet. Image acquisition was performed sequentially, with each microchannel perfused and imaged individually for the full experimental duration before proceeding to the next channel. During blood perfusion, time-lapse fluorescence imaging was performed using a 20 × objective (NA 0.7) in a loop sequence. Each loop lasted 20 seconds: all eight images were acquired within 10 seconds, followed by a 10-second pause before the next loop began. A total of 30 images per position were acquired during the perfusion. From the acquired images, the platelet-covered area was calculated as the ratio between platelet area and total image area (*A*ₚₗₜ/*A*ₜₒₜ) as a function of time, and the area under each curve (AUC) was calculated and expressed as *A*ₚₗₜ/*A*ₜₒₜ·s representing a cumulative measure of platelet–surface interaction over the full perfusion time. The rate of change in platelet accumulation and detachment was expressed as Δ *A*ₚₗₜ/*A*ₜₒₜ and was determined by computing the mean time derivative of *A*ₚₗₜ/*A*ₜₒₜ, providing an estimate of thrombus growth stability and detachment events. From this latter was calculated the velocity growth of platelet adhesion as mean of the derivatives points, and number of platelet detachments events as frequency of negative number in the derivatives curves.

#### Images Processing

Image processing was performed with custom Python scripts. Image stacks were split into frames, the background was corrected by subtracting the first frame and a threshold was applied to remove noise, after which binary images were generated for platelet deposition overlays. *A*ₚₗₜ/*A*ₜₒₜ was calculated as the fraction of positive pixels and extracted frame by frame to obtain time-resolved deposition curves.

#### Statistics

To evaluate differences in platelet accumulation (*A*ₚₗₜ/*A*ₜₒₜ) over time, a two-way repeated measures ANOVA was first performed with factors *time* and *group*. To further quantify these differences, the area under the curve (AUC) was calculated for each replicate and compared among groups by one-way ANOVA with Dunnett’s post hoc test, or by the Friedman test in the case of a nonparametric distribution, using the control as the reference. All analyses were performed in Prism 10.

## Results

### 2PP Microchannel Masters

Both the micropatterned bottom layer master and the ramped top layer master of the microchannel closely matched their design dimensions, with measurement deviations consistently below 1 *µ*m and low standard deviations, confirming reproducibility (Online Resource 1, Figs. S8–S10; Table S1 and Table S2).

### Computational Simulation of Microchannel and Microstructures

**Fig. 3:**
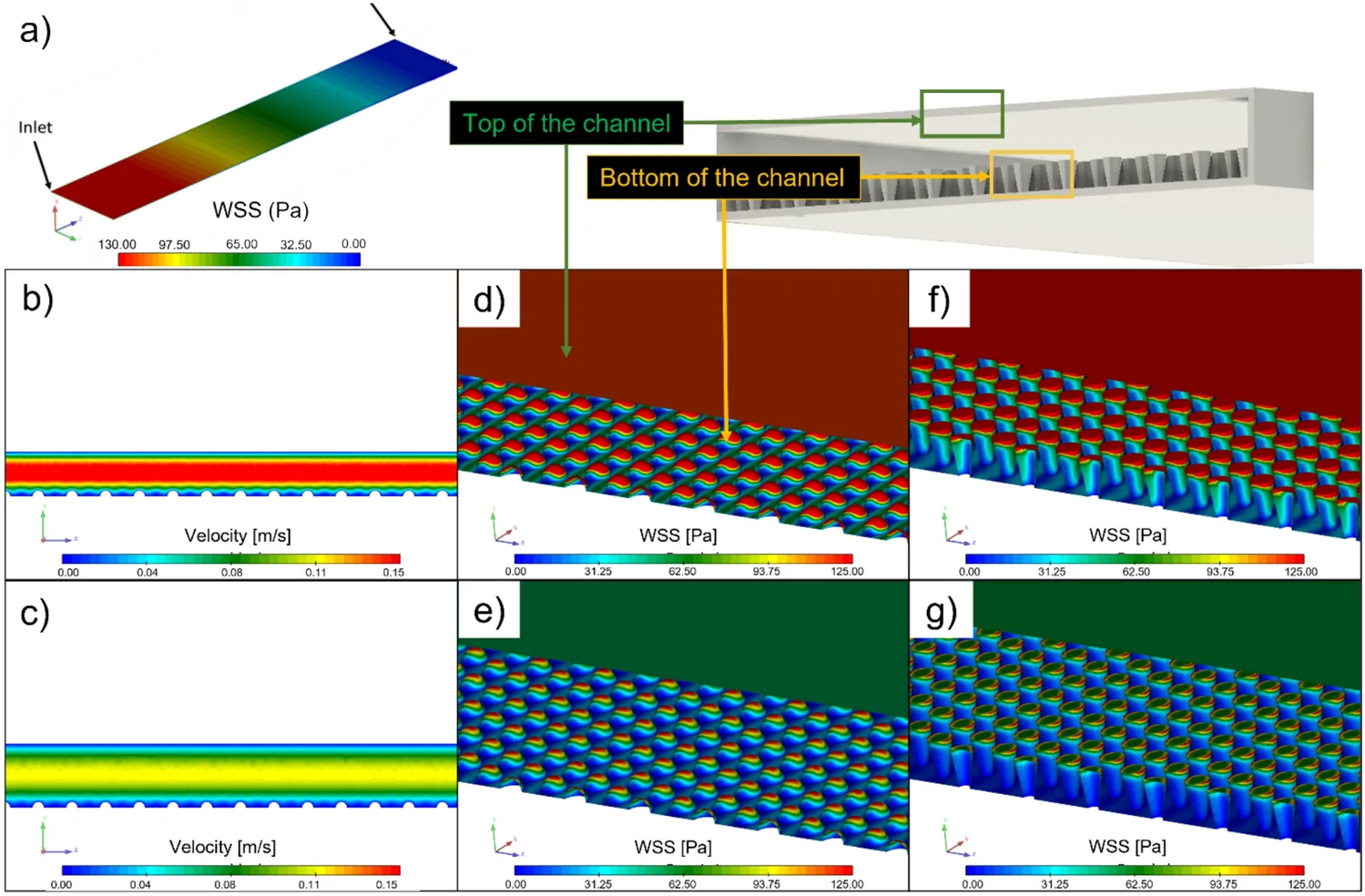
**a** WSS distribution within the channel with a blood velocity of 0.6 mL/min; **b** large hemispheres at 20 *µ*m–side view; **c** large hemispheres at 30 *µ*m side view; **d** large hemispheres at 20 *µ*m–isometric view, red uniform area is the channel ceiling above the structures on the ground; **e** large hemispheres at 30 *µ*m–isometric view, green uniform area is the channel ceiling above the structures on the ground; **f** large cones at 20 *µ*m–isometric view, red uniform area is the channel ceiling above the structures on the ground; **g** large cones at 30 *µ*m–isometric view, green uniform area is the channel ceiling above the structures on the ground. The scheme above 3f shows the isometric view of a channel section indicating the top and the bottom of the channel. The sketch is not in scale

WSS distribution in the channel is shown in Fig. 3a. The boundary layer had a constant thickness of 32.8% relative to channel height (Online Resource 1, Fig. S5). In all regions, microstructures remained within the boundary layer and did not impede flow (Online Resource 1, Fig. S6). The highest WSS values occurred at a microchannel height of 20 *µ*m, at the top of the microstructures, as shown in Fig. 3b and c. Figure 3d vs Fig. 3e and f vs 3 g illustrate the increase in WSS with higher flow velocities. The lowest WSS values were observed at the end of the microchannel, at a height of 112 *µ*m. The smallest microstructures, for both *C* and *H*, consistently produced the highest WSS values, reaching peak values of ~ 300 Pa for hemispheres and ~ 280 Pa for cones. This trend was observed across all operating points, with hemispheres (Fig. 3d) yielding slightly higher WSS values than cones (Fig. 3f). In cones, the WSS peaks were localized at the edges of the upper region, whereas in hemispheres they were concentrated at the center of the upper region.

In Fig. 3d–g, the WSS distribution is displayed at the top and bottom of the microchannel. At the top, the profile is smooth, reflecting a constant WSS value, whereas the microstructures at the bottom induce a heterogeneous WSS distribution with the highest peak values at the top of C and H and the lowest peaks at the ground, reaching values clearly below the smooth conditions. For example, at 30 *µ*m channel height the 93 and 60% of the surface analyzed are exposed to WSS magnitude lower than that experienced on the top of the channel, for LC and LH respectively. These results are consistent for all the simulations performed yielding average values of ~94% and ~58% respectively for C and H of the area in which WSS is lower than the value on the top of the channel. A reason for this is the geometry; cones hinder the flow more than hemispheres, causing a reduction of the velocity in the areas near them, thus a smaller WSS distribution.

### Platelet Adhesion

**Fig. 4:**
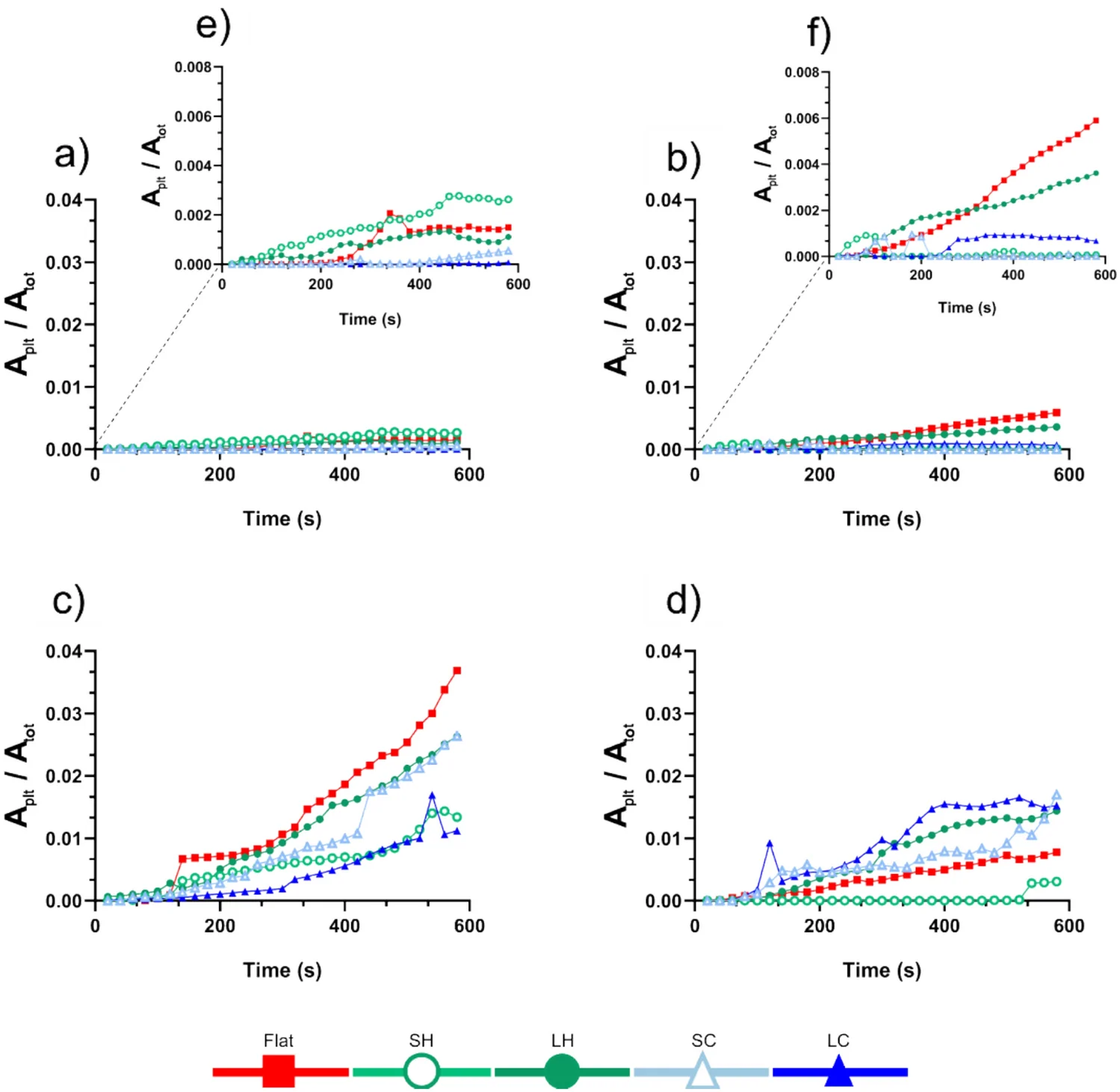
Ratio of platelet area coverage over time, (*A*ₚₗₜ/*A*ₜₒₜ): **a** 16; **b** 49; **c** 114; and **d** 130 Pa. **e** and **f** Plots on a magnified scale to better appreciate the trend lines. Six donors were used in independent experiments for each WSS condition and surface type. Standard deviations are not shown for clarity, as platelet attachment and detachment under flow conditions produced high variability

**Fig. 5:**
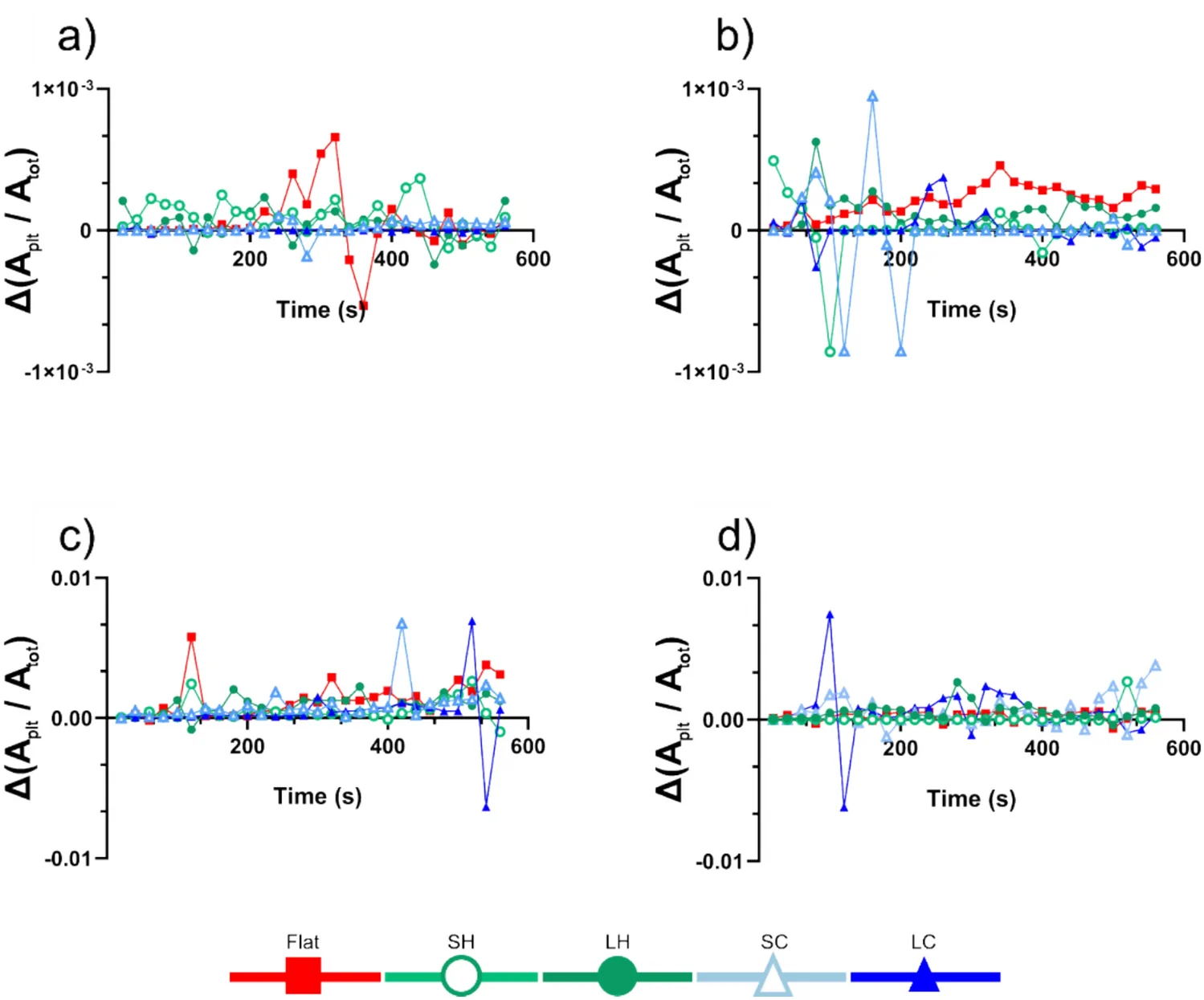
Mean derivatives of the ratio of platelet area coverage curves over time, Δ (*A*ₚₗₜ/*A*ₜₒₜ): **a** 16; **b** 49; **c** 114; **d** and 130 Pa. Six donors were used in independent experiments. Standard deviations are not shown for clarity, as platelet attachment and detachment under flow conditions produced high variability

Figure 4a and b displays *A*ₚₗₜ/*A*ₜₒₜ respectively at 16 and 49 Pa. Values remained below 1% by the end of the experiment (600 sec). In contrast, at 114 and 130 Pa in Fig. 4c and d, *A*ₚₗₜ/*A*ₜₒₜ increased, reaching 2–4% and 1–2% respectively. No statistically significant differences in *A*ₚₗₜ/*A*ₜₒₜ were observed among the micropatterned samples and the flat reference under any of the tested conditions. However, the trends indicated different biological behaviors across the samples. In Table 1, AUC values referring to the curves shown in Fig. 4 are summarized. SC (0.07 and 0.07 *A*ₚₗₜ/*A*ₜₒₜ·s) and LC (0.01 and 0.27 *A*ₚₗₜ/*A*ₜₒₜ·s) had very low AUC values at 16 and 49 Pa. SH shows high accumulation at 16 Pa (0.86 *A*ₚₗₜ/*A*ₜₒₜ·s) and a low value at 49 Pa (0.08 *A*ₚₗₜ/*A*ₜₒₜ·s). LH and the flat surface had AUC values more than sevenfold higher than the conical micropatterned surface at 16 Pa (0.42 and 0.45 *A*ₚₗₜ/*A*ₜₒₜ·s) and more than threefold higher than all the other samples at 49 Pa (1.07 and 1.33 *A*ₚₗₜ/*A*ₜₒₜ·s). At 114 Pa, all surfaces exhibited increased AUC values. SC, LC, and SH had the highest increase, but LH and the flat surface still had the highest AUC values. At 130 Pa, SC, SH, LH, and the flat surface experienced a decrease of 33, 95, 34, and 73%, respectively, while LC was the only surface showing an increase. Standard deviations were considered in the statistical analysis but not shown graphically (Figs. 4 and 5), as attachment and detachment events occurred at different time points across the 6 independent experiments, resulting in large deviations that obscure clear visualization.

**Table 1:** Area under the curve (AUC) of area covered by platelets over time for all surfaces (SC, LC, SH, LH, flat) at four WSS zones (16, 49, 114, 130 Pa), standard deviation (±), *N* = 6 donors for each WSS condition and surface type. Higher values indicate a greater overall platelet accumulation over 600 seconds

Area under the curves (*A*ₚₗₜ/*A*ₜₒₜ·s)						
WSS (Pa)		SC	LC	SH	LH	Flat
16	Mean	0.07	0.01	0.86	0.42	0.45
	Std. deviation	0.13	0.01	1.87	0.74	0.95
49	Mean	0.07	0.27	0.08	1.07	1.33
	Std. deviation	0.17	0.62	0.14	2.03	2.8
114	Mean	5.00	2.37	3.25	6.03	7.52
	Std. deviation	9.18	3.32	4.66	9.14	9.44
130	Mean	3.35	5.17	0.15	3.96	2.03
	Std. deviation	8.09	9.67	0.30	5.98	2.45

Figure 5 illustrates the rate of *A*ₚₗₜ/*A*ₜₒₜ change plotted against time. Positive values indicate platelet aggregates are growing, while negative values correspond to platelet aggregates detaching from the surface.

At 16 Pa (Fig. 5a), the oscillatory trend was observed especially for SH and the flat control, which exhibited faster platelet adhesion compared to the other micropatterned surfaces (Table 2). At 49 Pa, Fig. 5b, fluctuations were more pronounced for SC, LC and SH which showed a high frequency of detachments (respectively, 4, 11, 8 drops, Table 3). By contrast, the flat control and LH remained consistently positive, reflecting a faster (Table 2), stable, and continuous platelet attachment (Table 3). At low shear (16–49 Pa), conical surfaces (SC, LC) exhibited minimal growth and frequent detachment while flat surfaces promoted fast and stable adhesion at medium-to-high shear. At 114 and 130 Pa, Fig. 5c and d, fluctuations were larger in amplitude (note that a different y-axis range). At 114 Pa, the flat surface showed the highest growth velocity (Table 2) however, the number of detachment events remained low for all the samples (0–2). At 130 Pa, SC, LC, and LH showed a higher growth rate, but only SC and LC showed frequent detachments (7–9 events, Table 3), suggesting unstable thrombus formation. The flat surface grew more slowly and presented an intermediate number of detachments. SH showed the lowest growth related to a low number of detachments.

**Table 2:** Mean growth velocity of platelet adhesion, expressed as the average derivative (d *A*ₚₗₜ/*A*ₜₒₜ/dt) of the area coverage curves between 0 and 600 s. Values are intended to be multiplied by 10^−2^. Positive values indicate average platelet attachment. Data highlight differences in adhesion kinetics across surface patterns and shear zones

Growth velocity of *A*_plts_/*A*_tot_ (*10^−2^) [min^−1^]					
WSS [Pa]	SC	LC	SH	LH	Flat
16	0.12	0.02	0.56	0.24	0.32
49	0.00	0.14	0.01	0.77	1.26
114	5.65	2.40	2.87	5.51	7.91
130	3.64	3.27	0.66	3.08	1.62

**Table 3:** Number of platelet detachment events detected as negative derivative values within the experimental duration (600 seconds, 30 time points). Higher counts correspond to lower thrombus stability. The table compares how frequently thrombus destabilization occurs depending on surface microtopography and shear zone

# Detachments in 10 minutes					
WSS [Pa]	SC	LC	SH	LH	Flat
16	2	5	5	9	7
49	4	11	8	0	0
114	0	1	2	1	1
130	9	7	1	2	5

## Discussion

Surface micropatterning has been shown to reduce platelet adhesion under static and low WSS conditions, comparable to stagnant zones in LVADs [32]. For very high WSS, such as those investigated in this study (16, 49, 114, and 130 Pa), a significant knowledge gap remains, which is addressed here for the first time. In addition, this work introduces a highly precise manufacturing strategy together with previously unexplored micropattern geometries. A sophisticated microfluidic platform capable of measuring platelet deposition at four supraphysiological WSS levels on different micropatterned surfaces was developed, including hemispheres and reverse cones in two size ranges, small (1–3 *µ*m) and large (3–9 *µ*m), fabricated via 2PP and nanoimprinting. In this work, we extend 2PP capabilities by fabricating a perfectly smooth, non-planar ramp combined with high-resolution micropatterning over a large-area substrate. Collectively, this approach demonstrates continuous large-area 2PP masters at the ≈ 10^2^ mm^2^ scale, integrating macro-scale functional geometries with micrometer-scale surface features. To the best of our knowledge, such large, continuous, and hydrodynamically functional 2PP masters for NIL-based microfluidic replication have not been demonstrated at this scale. Furthermore, nanoimprinting lithography was adapted using a residual-layer-free MIMIC strategy to replicate micropatterns directly onto glass substrates forming the channel floor within vertically confined microfluidic geometries. This configuration decouples master fabrication from biocompatible experimental materials and enables large-area replication of high-resolution floor-integrated microstructures, representing a novel implementation of NIL for platelet-flow microfluidic platforms.

Platelet adhesion was governed by the combined effects of surface microtopography and local WSS. By evaluating cumulative platelet accumulation (AUC), adhesion kinetics, and detachment events, distinct surface-dependent behaviors emerged.

Unlike static or low-shear conditions, micropatterning did not consistently outperform flat surfaces under supraphysiological WSS. At low WSS (16–49 Pa), conical surfaces (SC and LC) showed the lowest platelet adhesion, minimal growth, and moderate detachment. Small hemispheres (SH) exhibited higher adhesion at 16 Pa but switched behavior at 49 Pa. Large hemispheres (LH) behaved similarly to the flat surface. At higher WSS (114–130 Pa), adhesion increased for all surfaces, with 114 Pa acting as a threshold promoting stable adhesion. At 130 Pa, cones exhibited high AUC but also high detachment, consistent with shear-induced washout. SH showed minimal adhesion, whereas LH sustained stable adhesion and behaved most similarly to the flat surface.

Few studies have investigated platelet adhesion on micropatterned surfaces; however, they provide valuable insights into platelet–microstructure interactions depending on geometries, size, and flow shear rate. According to Gester et al. [18], platelets contact only the tips of microstructures (micropillars and mushroom-shaped microstructures) for an interspacing of 3 *µ*m, resulting in reduced contact with the foreign material compared to flat surfaces. In contrast, increasing microstructure interspacing and dimensions (larger than platelet size) leads to platelet trapping, stagnation, promotion of platelet activation, and fast growth of thrombus formation [3, 33, 34]. In our study, the distances between cones and hemispheres were 1 and 3 *µ*m for small and large dimensions, respectively. This suggests that platelet aggregates mainly reside on top of the microstructures and are therefore exposed to more frequent washout. Furthermore, increased microstructure height may induce stronger flow disturbances and potentially promote platelet accumulation; however, the microstructures used in this study remained within the boundary layer (Online Resource 1, Fig. S5) and therefore did not act as flow obstacles [8]. Pham et al. [19] showed that increasing microstructure height (2.7 vs. 2.5 and 0.9 *µ*m for hemispherical segment microstructures) reduced platelet adhesion due to a larger WSS gradient between the top and the base of the microstructures, which facilitates platelet washout. Consistent with this observation, LC, which are the tallest microstructures in our study, experienced the highest number of detachment events. In our simulations, a well-defined WSS gradient was observed along the height of the microstructures, with lower WSS values at the ground compared to the flat surface and peaks at the top reaching approximately 300 Pa.

However, at lower shear levels, microstructure shape rather than size dominated platelet adhesion behavior; indeed, conical structures performed better than all other samples at 16 and 49 Pa. At higher shear levels (114 and 130 Pa), adhesion did not follow a clear pattern. Notably, conical microstructures have not previously been experimentally tested in this context, likely due to fabrication challenges. However, theoretical models suggest that conical features are optimal for inducing superhydrophobicity and reducing drag [14], which may help explain the reduced platelet adhesion observed on cones at WSS below 114 Pa.

Importantly, platelet adhesion rate is not purely driven by microstructure geometry and sizes but is also influenced by the local flow field, shear rates and velocity gradients. Contrary to our results, He et al. [3] reported that high shear (about 26 Pa) resulted in lower overall platelet deposition due to the washout effect, while regions of lower shear and flow velocity showed higher accumulation. However, the highest shear tested in their study is close to our lowest applied shear, suggesting that in the presence of microstructures, the relationship between shear stress and platelet deposition is nonlinear. In our study, platelet adhesion increased at WSS > 49 Pa, consistent with shear-induced platelet activation and aggregation thresholds (50–90 Pa) [35, 36], which may promote platelet accumulation. Moreover, the increased platelet deposition is affected by the geometry of the microchannel. The narrowing of the microchannel induces higher flow velocities and shear stresses, which enhance shear-induced transport mechanisms. In particular, elevated shear rates promote platelet margination toward the wall, increasing near-wall platelet concentration and likelihood of adhesion [37]. Therefore, while microstructure geometry influences local interactions, the microchannel flow field contributes to the platelet adhesion through shear-enhanced margination and transport.

A limitation of this study can be attributed to the narrow channel geometry (20 *µ*m), which could exacerbate platelet adhesion through the marginalization effect. However, if this aspect dominated, a comparable increase in adhesion would be expected across all surface geometries in higher WSS regions. Instead, we observed geometry-dependent differences in surface coverage. The experimental system is complex, which represents both a limitation and a strength. The setup involves multiple variables that require careful control, including complex microstructure fabrication, channel height gradient, background fluorescence, and the high pressures within the system, together with the associated risk of clot formation. However, the same setup is highly adaptable: by increasing the channel height, different flow rates and WSS conditions can be tested, enabling systematic investigation of the thresholds at which surface topography or hydrodynamic effects become dominant.

Another limitation of this study is the use of whole blood diluted 1:1 with PBS to enable reliable fluorescence imaging of platelet adhesion under flow, as undiluted blood in microfluidic systems leads to early signal saturation. According to the literature, this level of dilution does not affect platelet functionality [31]. However, the dilution with PBS was applied consistently across all experiments, which resulted in an altered and non-physiological hematocrit, and the viscosity approximation used in this study may introduce uncertainty in the estimation of the reported WSS values. Measuring the hematocrit for each blood sample and diluting with plasma would further improve accuracy. Nevertheless, the primary focus of this work was on relative differences in platelet adhesion and detachment as a function of microtopography and WSS; future studies could benefit from adjusting whole blood with plasma to a fixed target hematocrit to achieve improved control over viscosity and hemodynamic parameters. In addition, this study is focused on the quantification of platelet aggregate formation, which limits the evaluation of other important contributors to platelet adhesion, such as blood proteins [38], particularly von Willebrand factor (vWF) [8], and how micropatterning may influence its behavior under high shear. Although the role of vWF has been extensively investigated, its function under supraphysiological WSS remains incompletely understood. Recent studies have reported that platelet activation decreases with increasing surface-bound vWF density under flow [39], a finding that contrasts with the acquired vWf syndrome observed in LVAD patients, which is characterized by reduced circulating vWF levels and an increased bleeding risk [1]. Moreover, many in vitro studies, including the present one, are limited to short-duration experiments compared to prolonged exposure to supraphysiological WSS performed in LVADs. Future studies will therefore focus on tuning microchannel geometries, using a constant and more physiological hematocrit, and performing a more comprehensive evaluation of blood protein behavior under extended flow conditions.

In conclusion, the data indicate that, under low WSS (< 49 Pa) conditions, conical microstructures exhibited lower platelet adhesion compared to the control surface. However, at higher WSS, platelet adhesion increased, suggesting platelet retention within the cone valleys. At the same time, the frequency of platelet detachment also increased, likely due to the heterogeneous WSS distribution generated by the sharp edges of the cones and the strong local WSS gradients between the top and ground of the cones (> 200 Pa). Furthermore, it was observed that small hemispherical features maintained lower platelet adhesion at WSS values greater than 16 Pa. This behavior is likely attributable to their morphological resemblance to the native endothelial surface and to their reduced dimensions, which differentiate their effect from that observed with larger hemispheres. These findings indicate that under WSS conditions relevant to LVADs, surface micropatterning does not eliminate platelet adhesion but can modulate it by the washout effects, shortening the time platelets spend adhering to the surface and limiting sustained thrombus growth. Therefore, microtopography may represent a promising passive strategy to limit excessive thrombus development in high-shear blood-contacting devices and warrants further investigation.

## Supplementary Information

Below is the link to the electronic supplementary material.

Supplementary file1 (PDF 1000 KB)

## Data Availability

Part of the data supporting the findings of this study is included in the article’s supplementary materials “Online Resource 1.” Additional data are available upon reasonable request from the corresponding author, M. Bonora.
