# Supplementary material for: The Influence of Micropatterned Surfaces on Platelet Adhesion to Reduce the Risk of Thrombus Formation in Left Ventricular Assist Devices
Source: Ann Biomed Eng. 2026 Mar 27;54(11):4082–93. doi: 10.1007/s10439-026-04108-7 (PMC13694399; doi:10.1007/s10439-026-04108-7)
Supplement: Supplementary file 1 [file 10439_2026_4108_MOESM1_ESM.pdf]

Supplementary Information (SI)

Annals of Biomedical Engineering

Title

The influence of micropatterned surfaces on platelet adhesion to reduce the risk of thrombus formation in left ventricular assist devices

Author information

Marta Bonora[1,2], Stjepan Perak[3], Sonja Kopp[4], Josu Garcia Morales[5], Richard Benauer[5], Markus Lunzer[3], Michael Mühlberger[4], Teresa Ruthmeier[6], Barbara Messner[6], Michael Bernhard Fischer[7,8], Francesco Moscato[1,2,9]

[1] Center for Medical Physics and Biomedical Engineering, Medical University of Vienna, Vienna, Austria;

[2] Ludwig Boltzmann Institute for Cardiovascular Research, Vienna, Austria;

[3] UpNano GmbH, Vienna, Austria;

[4] PROFACTOR GmbH, Steyr, Austria;

[5] Bionic surface technologies GmbH, Graz, Austria;

[6] Department of Cardiac and Thoracic Aortic Surgery, Medical University of Vienna, Austria, Vienna;

[7] Department of Transfusion Medicine and Cell Therapy, Medical University of Vienna, Vienna, Austria;

[8] Department for Biomedical Research, University for Continuing Education Krems, Krems, Austria;

[9] Austrian Cluster for Tissue Regeneration, Vienna, Austria;

Corresponding author: Marta Bonora marta.bonora@meduniwien.ac.at, ORCID: 0000-0003-3667-0965

1. Channel design

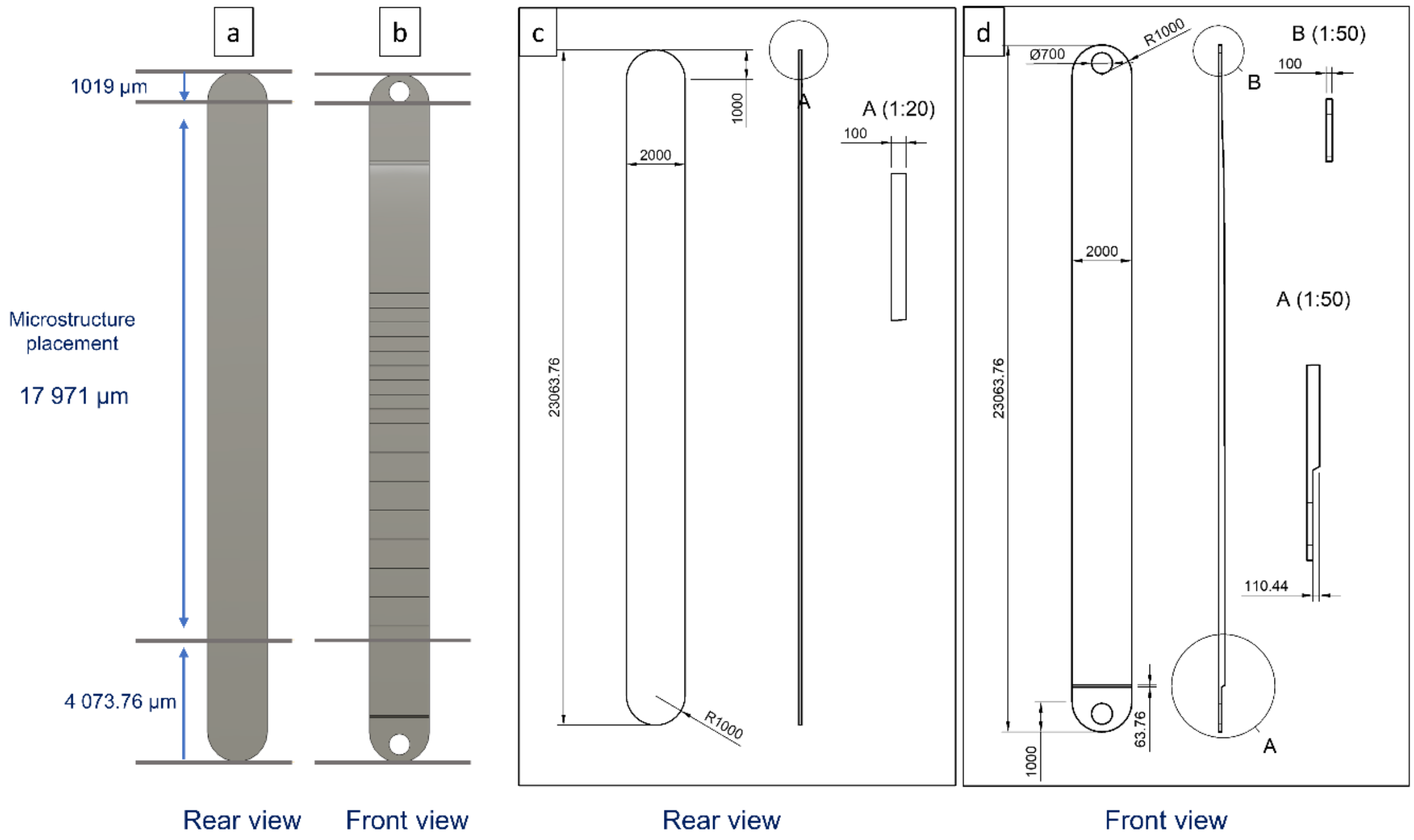

Fig. S1 CAD design of the microfluidic ramp used for nanoimprinting and device assembly. (a) Rear view showing the microstructure placement region. (b) Front view of the ramp. (c) Rear view with detailed dimensions and section A. (d) Front view with detailed dimensions and enlarged sections A and B. All dimensions are reported in µm.

2. Simulation

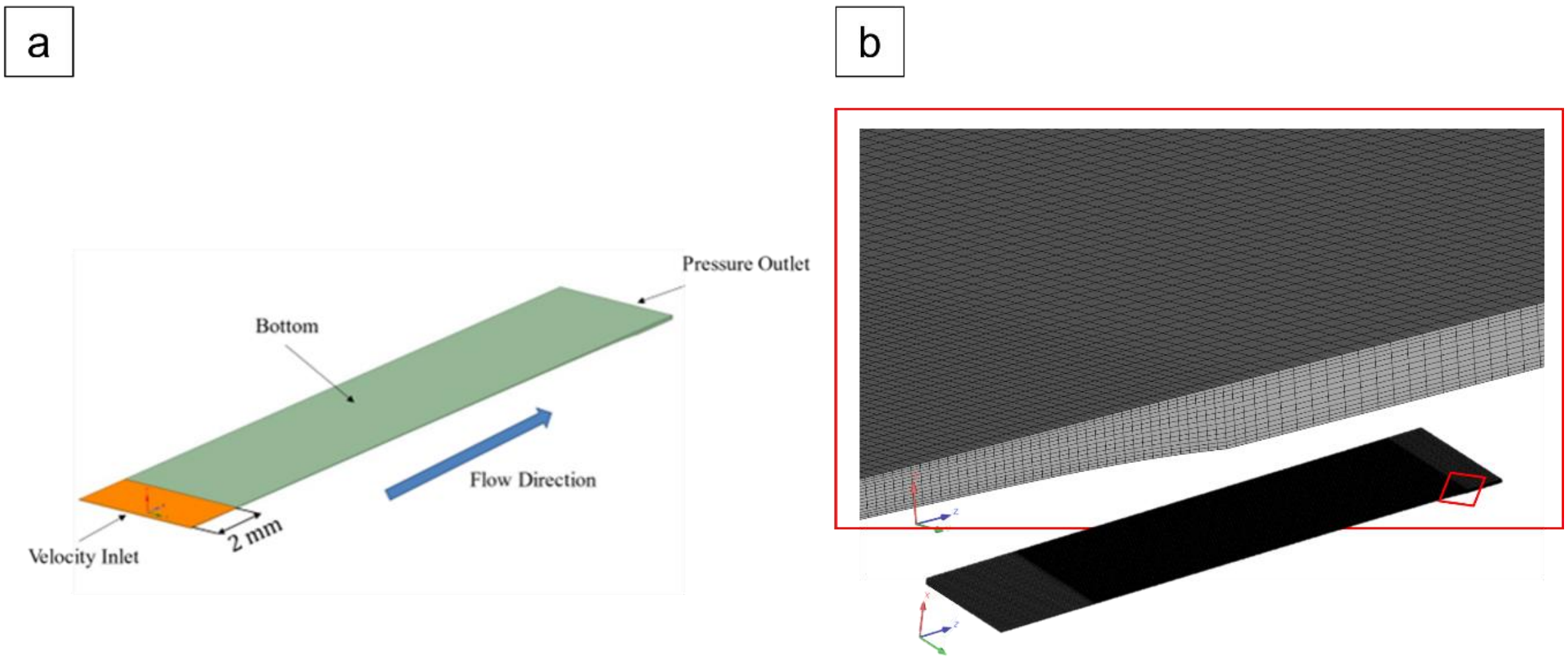

Fig. S2 (a) Design of the microchannel used for the simulation with the 2 mm long entrance added in orange; (b) Design mesh of the microchannel and its magnification on details

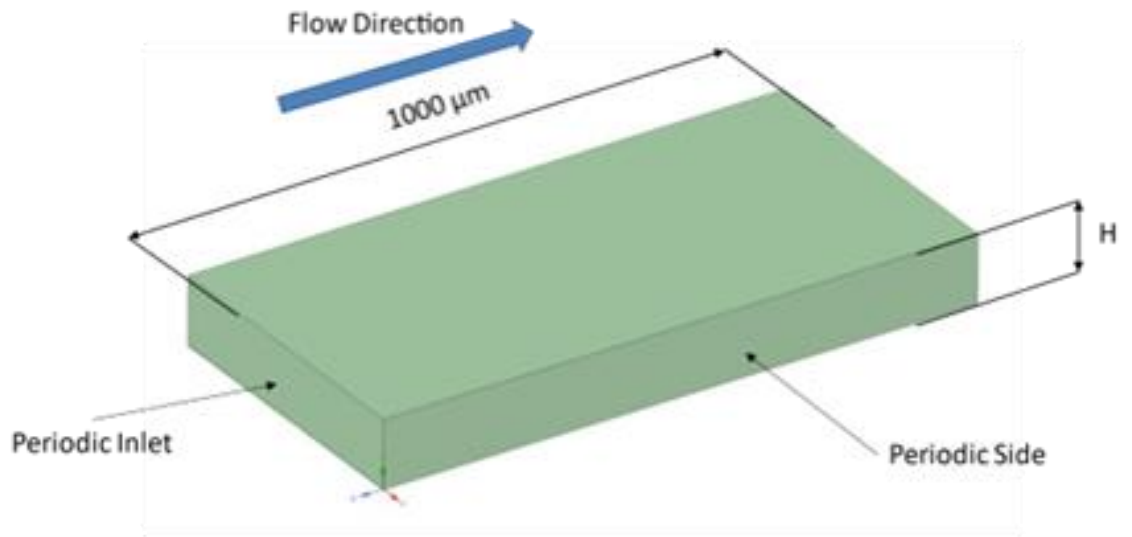

Fig. S3 Periodic domain, implemented with periodic boundary conditions

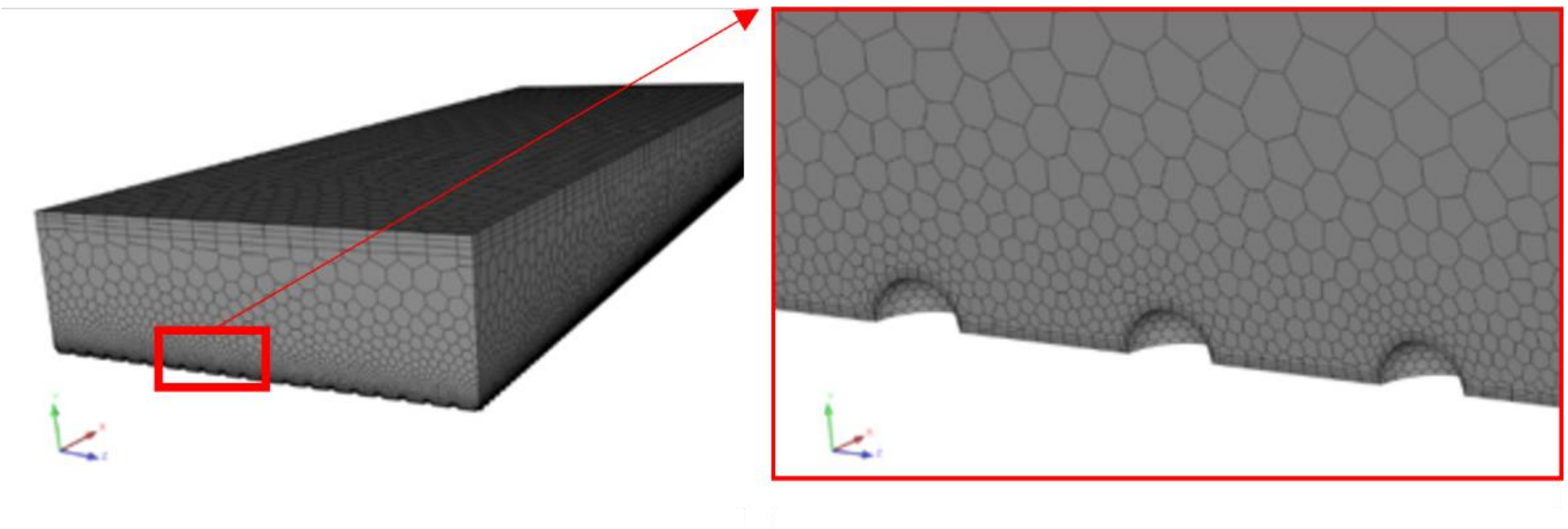

Fig. S4 Mesh of the of the microstructures; in magnification image: hemispheres at 112 µm height of the microchannel

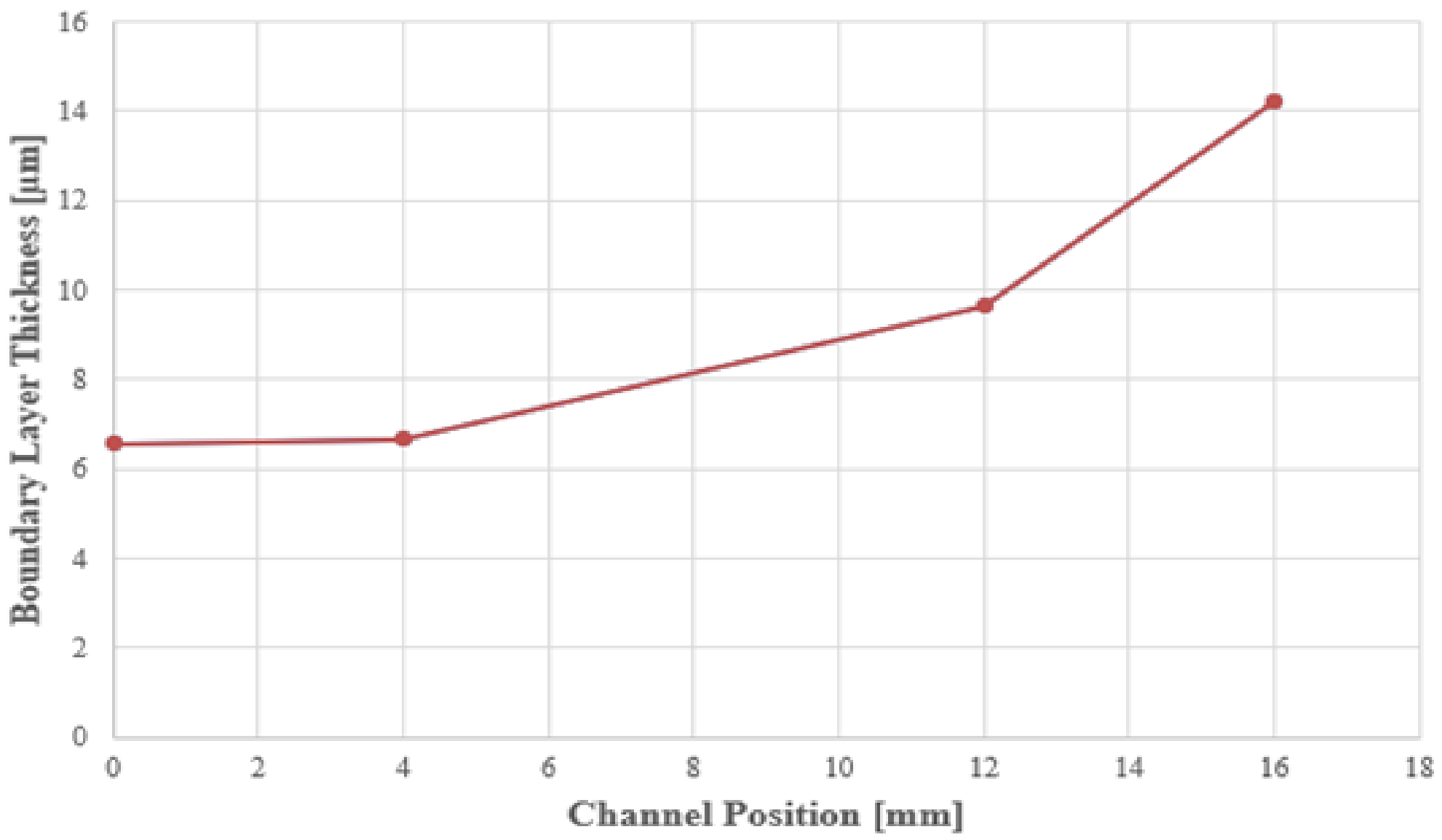

Fig. S5 Boundary layer analysis graph. The boundary layer thickness plotted with respect the length of the channel

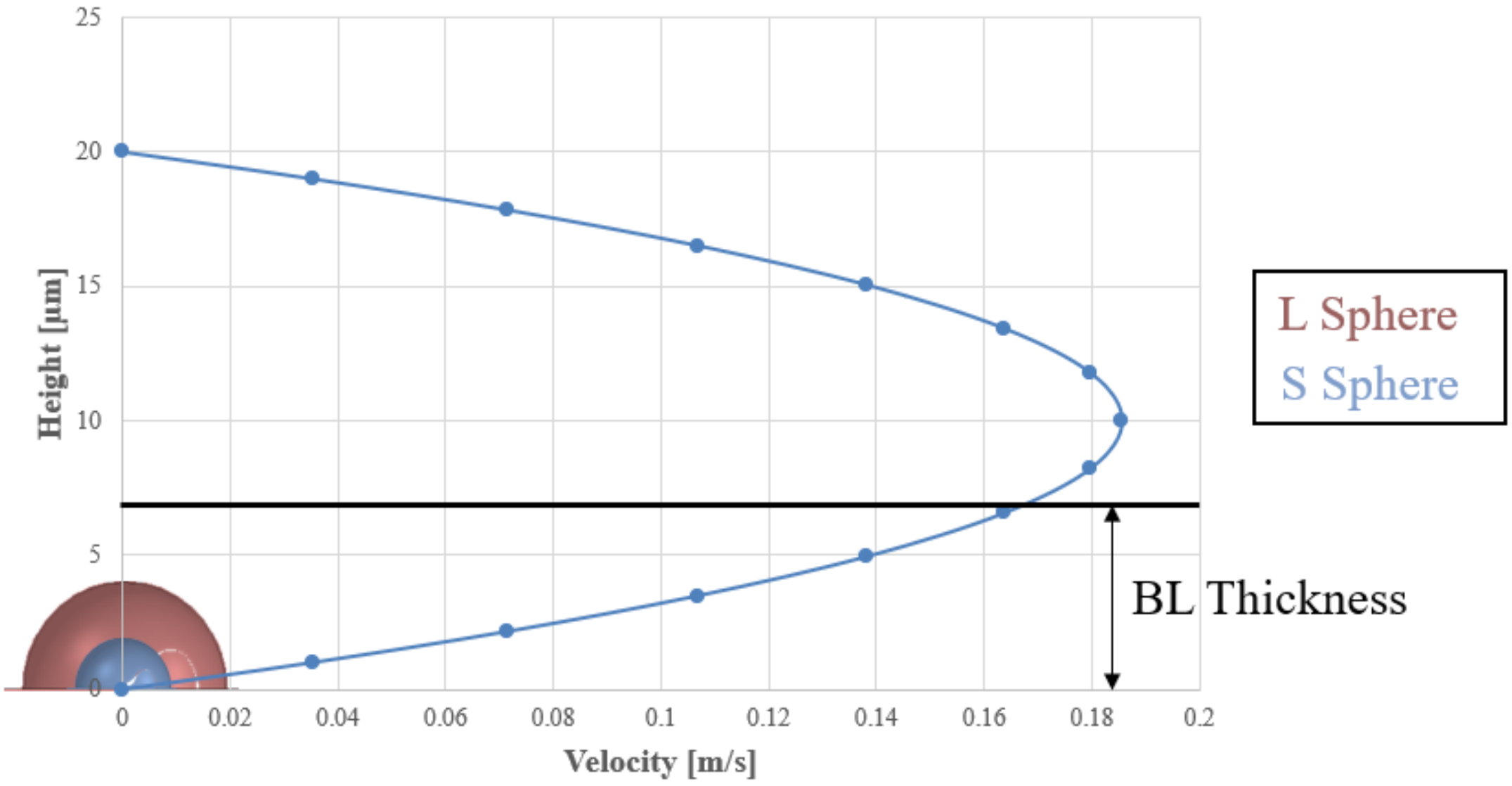

Fig. S6 Height of the microstructures (max 6 µm) plotted with respect the boundary layer thickness

3. Nanoimprinting

NIL [1], [2] is a very versatile replication technology for micro- and nanostructures. Starting from a master structure a stamp is created, which contains the negative surface topography of the master. The surface which should be structured is coated with a UV-curable material and the stamp is brought into contact with the surface. The liquid UV-curable resin fills the stamp cavities and as soon as this process is finished the material is hardened by UV-radiation. Then the stamp can be removed and be used for the next imprint. This process allows the fast and cost-effective replication of structures ranging from several nanometers to several 100 µm on areas up to several dm^2. A broad range of materials can be used and also complex structures can be replicated in a single imprinting step e.g., biomimetic structures [3]. Further, to improve throughput and reduce the risk of damaging the master, replication of the master itself by nanoimprinting was also explored. As shown in Fig. S6, a 2PP-printed master (right) was duplicated into five copies on a 150 mm glass wafer, enabling parallel fabrication of five top covers in a single process.

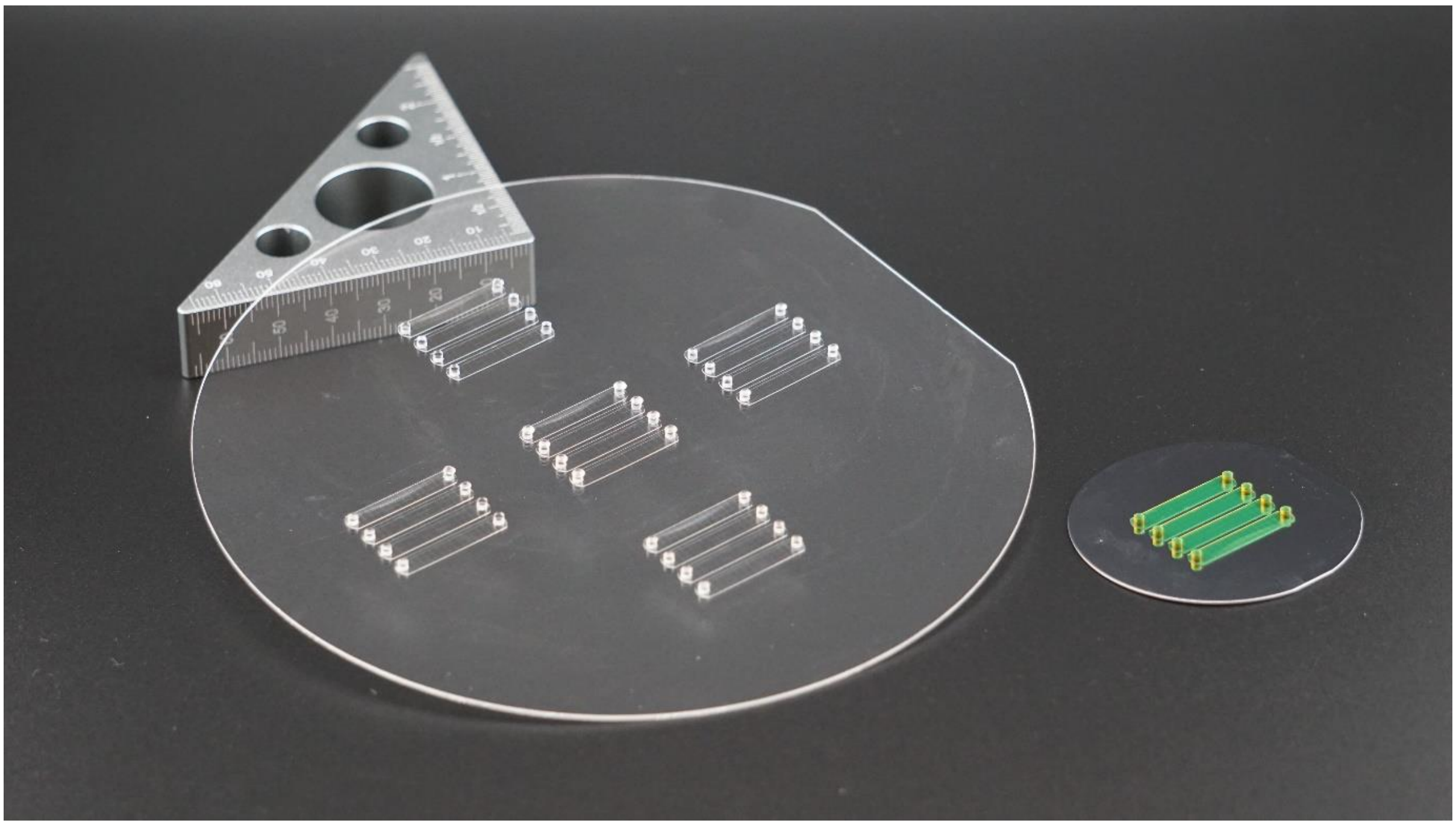

Fig. S7 2PP-printed master (right) was duplicated into five copies on a 150 mm glass wafer, enabling parallel fabrication of five top covers in a single process

4. 2PP microchannel masters

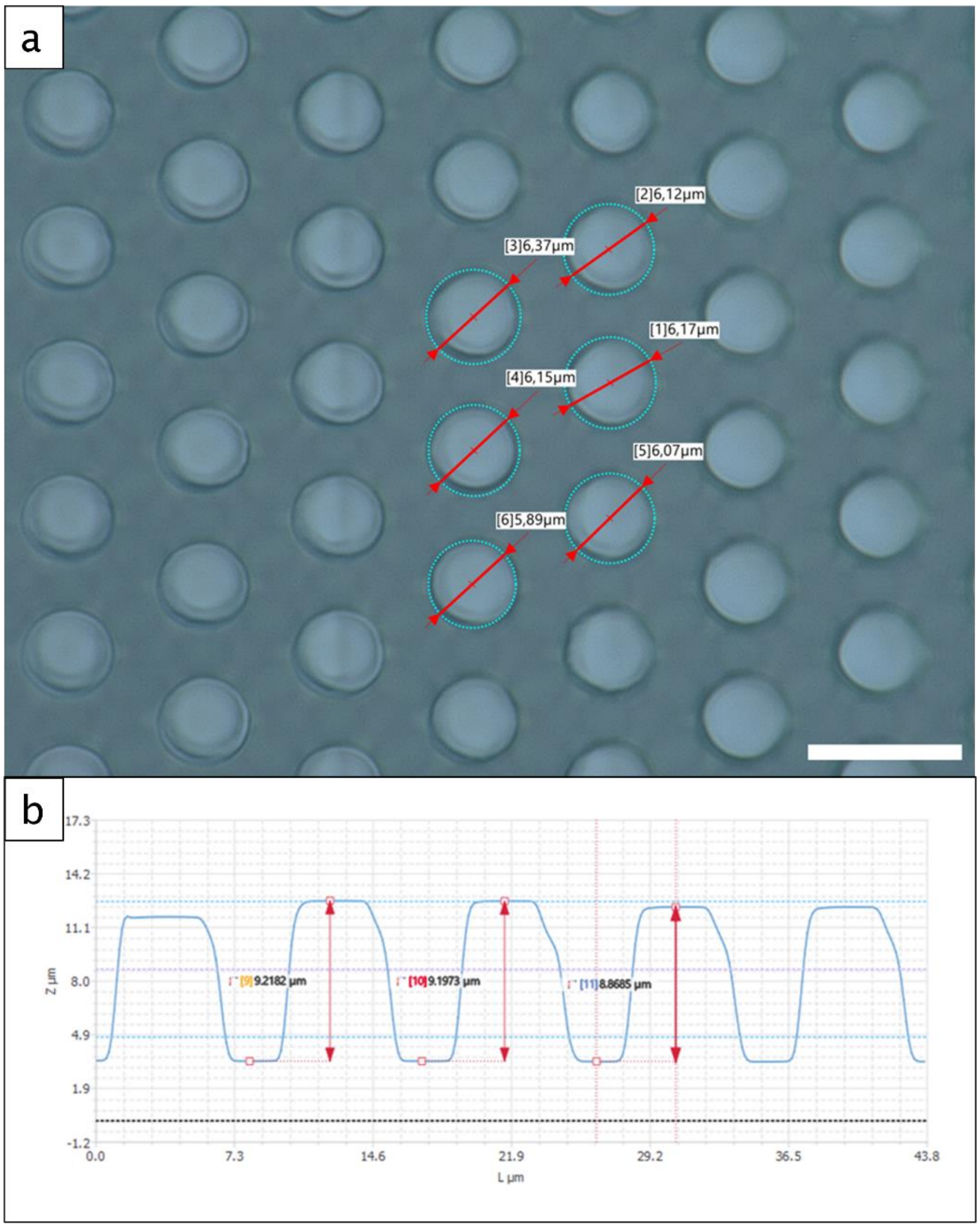

Fig. S8 a) Light microscopy image (Keyence VHX-7000) showing top diameters of reversed cones large (Scale bar: 10 µm. N=6), b) The height (N=3) was determined by measuring the distance between the top and bottom surfaces using optical profilometer (Sensofar S neox, Magnification 50x, N=3). Due to optical limitations to capture slopes above a certain threshold angle, the technique cannot resolve the side profiles and thus does not provide an accurate cross-sectional view. The mean diameter values can be found in the Table S1

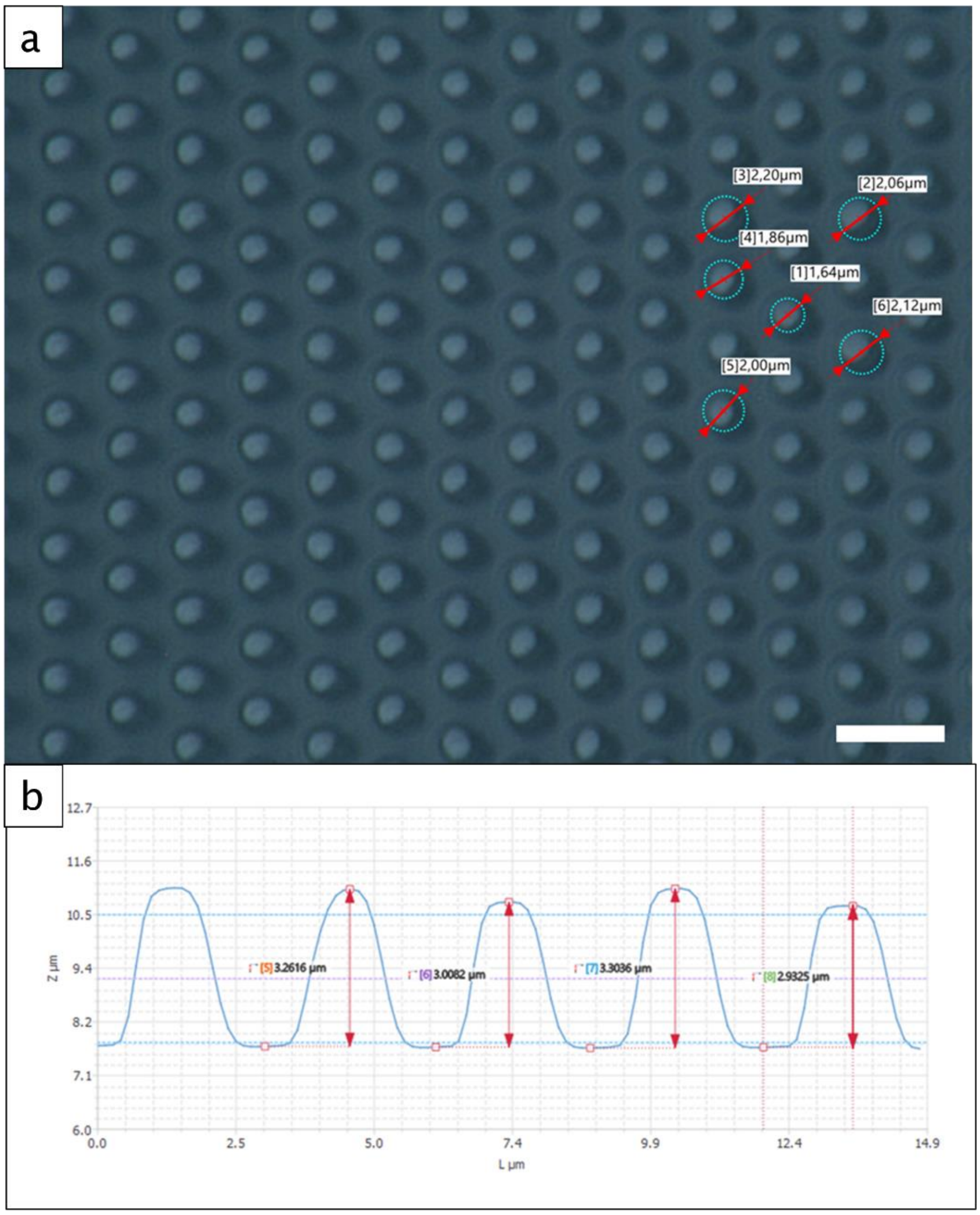

Fig. S9 a) Light microscopy image (Keyence VHX-7000) showing top diameter of reversed cones small (Scale bar: 5 µm. N=6), b) The height was determined by measuring the distance between the top and bottom surfaces using optical profilometry (Sensofar S neox, Magnification 50x, N=4). Due to optical limitations to capture slopes above a certain threshold angle, the technique cannot resolve the side profiles and thus does not provide an accurate cross-sectional view. The mean diameter values can be found in the Table S1

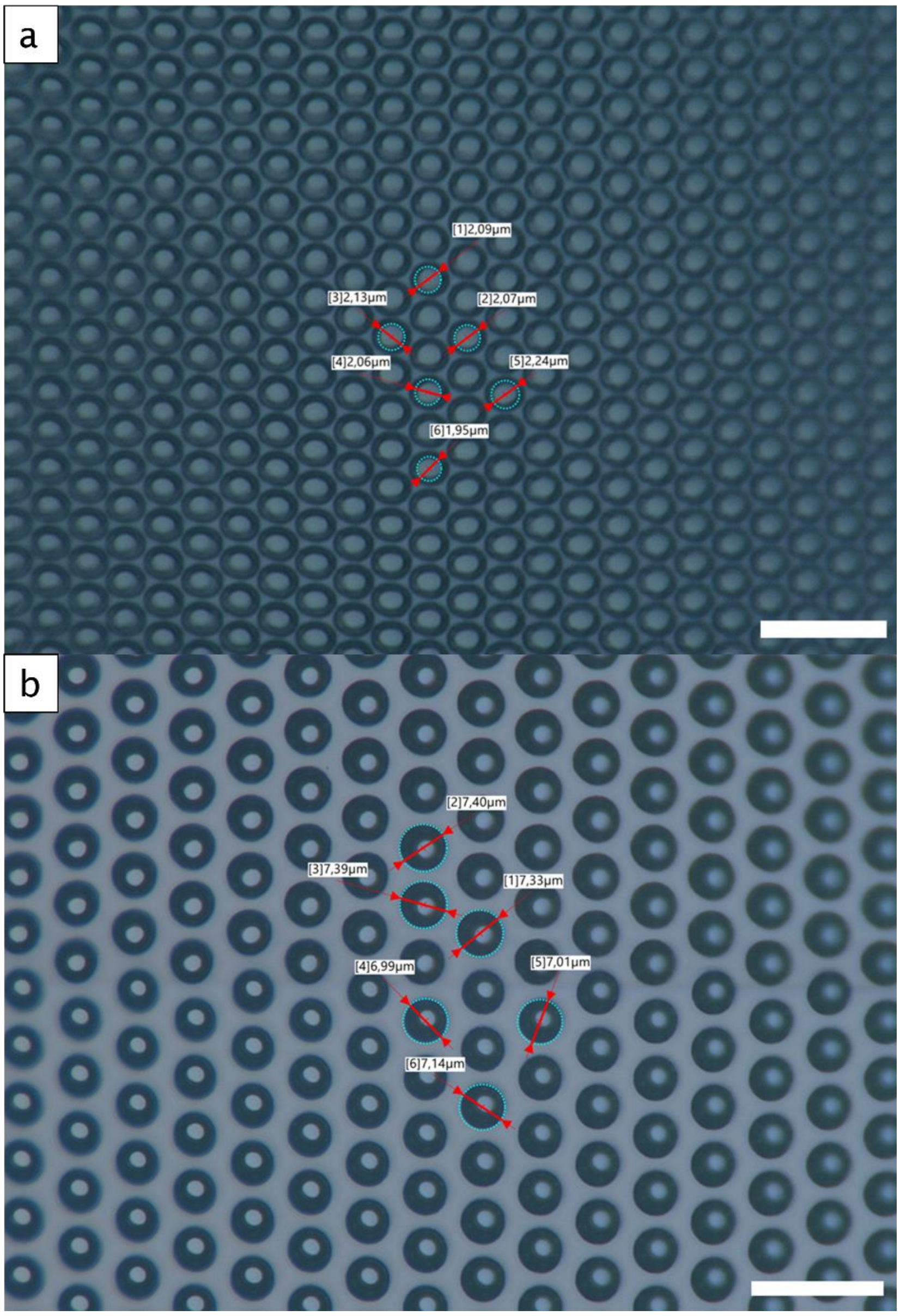

Fig. S10 (a) Light microscopy image (Keyence VHX-7000) showing diameter (N=6) of the hemispheres large. The mean diameter values can be found in the Table S1. Scale bar 20 µm. (b) Light microscopy image (Keyence VHX-7000) showing diameter (N=6) of the hemispheres small. The mean diameter values can be found in the Table S1. Scale bar 10 µm

Table S1 Mean top diameter and height measurements in µm of the reverse cones (C) microstructures including standard deviation (SD, N=6)

Reverse Cones master measurements [µm]				
			2PP 3D printed [µm]	
**Size**	Feature	Designed	Mean	SD
**Large (L)**	Top diameter	6	6.1	0.14
	Height	9	9.1	0.02
**Small (S)**	Top diameter	2	1.9	0.18
	Height	3	3.1	0.16

Table S2 Diameter measurements in µm of the hemisphere (S) microstructures including standard deviation (SD, N=6)

Hemispheres master measurements [µm]				
			2PP 3D printed	
**Size**	Feature	Designed	Mean	SD
**Large (L)**	Diameter	6	6.7	0.17
**Small (S)**		2	2.0	0.06

Top layer

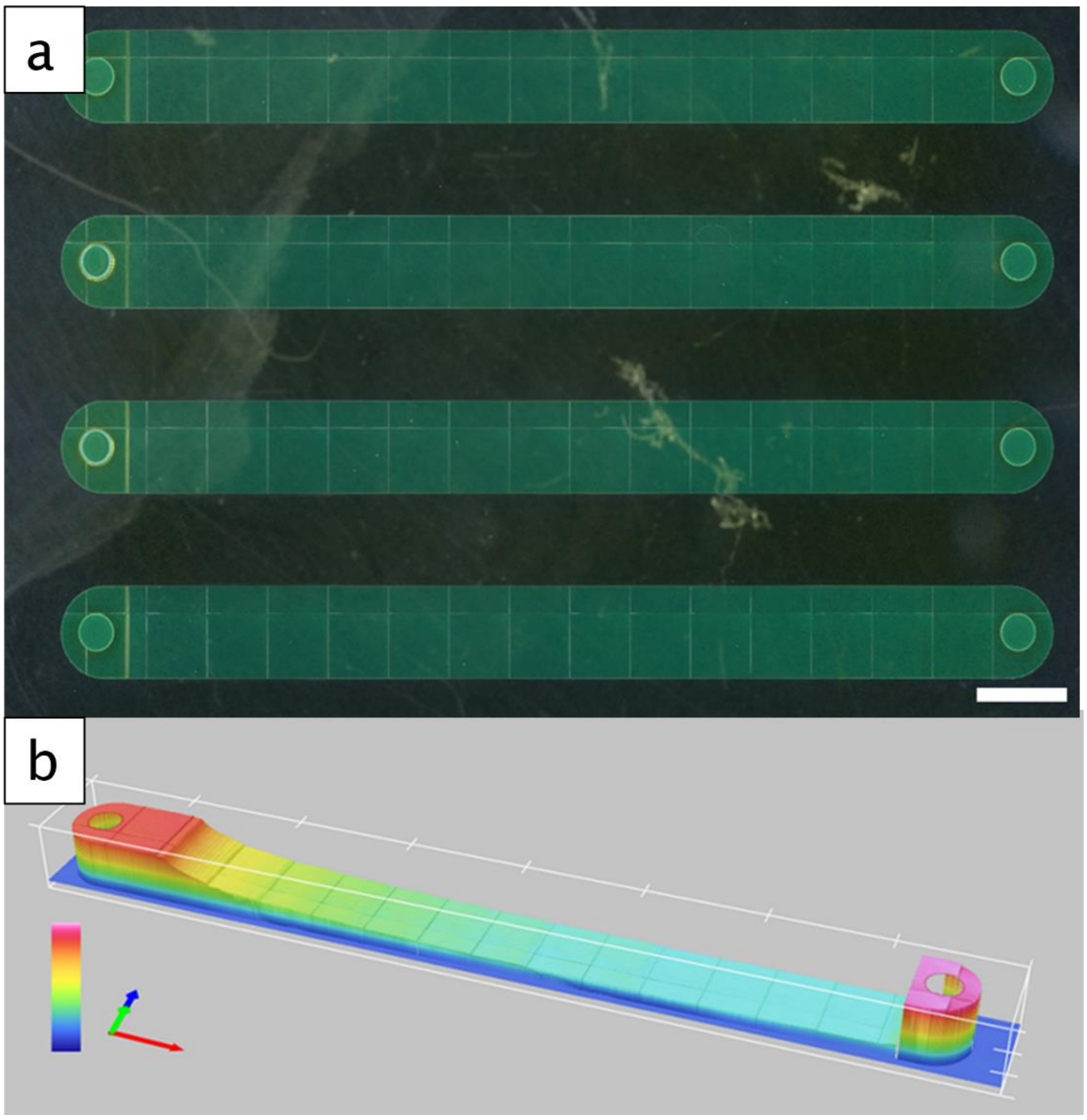

Fig. S11 a) Overview light microscopy image (Keyence VHX-7000) of a 2PP 3D printed master of the microchannel top layer ramps (negative), each 23 mm long, 2 mm wide and 0.45 mm high. Scale bar 2 mm. b) Optical profile of the ramped top cover channel master (Sensofar, S Neox, 50x)

Reference

[1] *Nanoimprint Lithography Technology and Applications*. 2022. doi: 10.3390/books978-3-0365-4481-6.

[2] H. Schift, “Nanoimprint lithography: An old story in modern times? A review,” *Journal of Vacuum Science & Technology B: Microelectronics and Nanometer Structures Processing, Measurement, and Phenomena*, vol. 26, no. 2, 2008, doi: 10.1116/1.2890972.

[3] M. Muehlberger, “Nanoimprinting of Biomimetic Nanostructures,” *Nanomanufacturing*, vol. 2, no. 1, 2022, doi: 10.3390/nanomanufacturing2010002.
